# Malaria and determinants of health: a scoping review of malaria vulnerabilities in Southeast Asia

**DOI:** 10.1186/s41182-025-00784-8

**Published:** 2025-08-04

**Authors:** Shahrul Azhar Md Hanif, Mohd Rohaizat Hassan, Nazarudin Safian, Rosnah Sutan, Alabed Ali A. Alabed, Muhammad Ridzwan Rafi’i, Siti Najiha Md Asari, Nurul Athirah Naserrudin, Rahmat Dapari

**Affiliations:** 1 Department of Public Health Medicine, Faculty of Medicine, Universiti Kebangsaan Malaysia, Jalan Yaacob Latif, Cheras, Kuala Lumpur, Malaysia; 2 University of Cyberjaya, Persiaran Bestari, Cyber 11, 63000 Cyberjaya, Selangor Malaysia; 3 Community Medicine Department, Faculty of Medicine, University of Cyberjaya, Persiaran Bestari, Cyber 11, 63000 Cyberjaya, Selangor Malaysia; 4 Health Services Research Center, Institute for Health Systems Research, National Institute of Health, Ministry of Health Malaysia, Shah Alam, Selangor Malaysia; 5 Faculty of Medicine and Health Sciences, Universiti Putra Malaysia, 43400 Serdang, Malaysia; 6 Integrated Dengue Research and Development, Faculty of Medicine and Health Sciences, Universiti Putra Malaysia, 43400 Serdang, Malaysia

**Keywords:** Malaria, Vulnerabilities, Risk, Determinant of health, Health inequities

## Abstract

**Introduction:**

Malaria continues to pose a considerable public health challenge in Southeast Asia, necessitating control efforts that consider region-specific vulnerabilities. Although global risk factors are well-documented, the interplay of social determinants of health in shaping malaria vulnerability within SEA populations is less thoroughly explored.

**Objective:**

This scoping review aims to determine elements influencing vulnerabilities towards malaria in SEA populations. In addition, this study seeks to explore how various social determinants of health contribute to the increased malaria vulnerabilities in this region.

**Method:**

This review adhered to the PRISMA-ScR guidelines. A systematic literature search was performed in PubMed, Scopus, and Web of Science from October to November 2024, focusing on peer-reviewed, open-access quantitative studies published from 2014 to 2024. Studies that addressed the risk or vulnerability of malaria in SEA populations were included. Multiple reviewers independently conducted screening, data charting, and thematic synthesis.

**Result:**

Twenty-two studies met the inclusion criteria, encompassing various malaria-endemic settings in SEA, including cross-border regions and migrant populations. The findings were synthesized using a social determinants of health lens, resulting in five thematic domains: biological influences, demographic and socioeconomic parameters, built and lived environments, behaviour and practices, and access to healthcare services and information.

**Conclusion:**

The vulnerability of SEA population to malaria is multifaceted and deeply entrenched in a web of complex, interrelated factors. Effective control requires region-specific, multi-sectoral strategies that address these vulnerabilities through targeted interventions, strengthened health systems, and equitable public health policies.

**Supplementary Information:**

The online version contains supplementary material available at 10.1186/s41182-025-00784-8.

## Introduction

Malaria continues to be a significant public health concern in Southeast Asia (SEA), despite considerable advancements in control measures over the last decade [1]. The region persists in facing considerable differences in malaria incidence attributable to variables including population movement, vector resistance, and inconsistent execution of control methods [2, 3]. Countries such as Vietnam, and Cambodia have significantly reduced transmission rates, whilst others, including Myanmar, Laos and Indonesia, continue to experience a substantial disease burden [4, 5]. Recent reports indicate a resurgence of malaria in western Thailand near the Myanmar border, along with new cases in previously low-incidence areas, highlighting the changing and inconsistent landscape of malaria susceptibility in the region [4, 6]. Despite recent years experiencing a deceleration in advancements attributed to drug resistance (particularly to artemisinin), difficulties in reaching distant communities, and geopolitical instability, SEA documented a 77% decrease in malaria cases, from 22.8 million in 2000 to 5.2 million in 2022 [2, 5]. Nevertheless, the region had more than 8000 malaria-related fatalities in 2022 and constituted 18% of the worldwide malaria burden, highlighting the enduring vulnerabilities there [1].

Malaria control strategies in SEA have focused on a combination of vector control and treatment protocols. Insecticide-treated bed nets (ITNs), indoor residual spraying (IRS), and extensive distribution of artemisinin-based Combination Therapies (ACTs) are fundamental to national malaria programmes [5, 7]. Countries including Cambodia, Myanmar, and Laos have augmented ITN distribution and IRS implementation, particularly in high-risk rural and forested areas [2]. Global health organizations, such as the World Health Organization, endorse these initiatives by prioritizing targeted interventions for at-risk populations. The implementation of rapid diagnostic tests (RDTs) has markedly augmented diagnostic capabilities in remote regions, facilitating expedited treatment and improving surveillance [8]. Regional cooperation focused on standardizing treatment and enhancing cross-border surveillance have been crucial in combating the proliferation of drug-resistant malaria [9, 10]. Furthermore, in order to reach people in remote locations and educate them on how to avoid malaria, many SEA countries have adopted more comprehensive strategies, like community engagement and community health workers [11, 12].

Notwithstanding these initiatives, vulnerabilities persist significantly, especially within marginalized groups. The transmission of malaria in Southeast Asia is significantly affected by a confluence of geographical, environmental, and socioeconomic factors [13, 14]. The region’s tropical climate, marked by elevated temperatures, humidity, and precipitation, creates optimal circumstances for the reproduction of Anopheles mosquitoes [15, 16]. Economic inequalities intensify health risks, as disadvantaged groups frequently lack access to adequate diagnoses, treatment, and preventive measures [17]. Rural communities encounter further obstacles owing to inadequate healthcare facilities and protracted access to care [17, 18]. Migrant workers, refugees, and people residing in conflict-affected areas face heightened vulnerability due to overcrowded living conditions and interrupted health services [17]. The lack of awareness regarding malaria symptoms and prevention strategies exacerbates delays in diagnosis and results in suboptimal treatment outcomes [19]. Control efforts are also made more difficult by the emergence of drug-resistant strains of malaria and environmental factors such as deforestation and climate change [20].

Although malaria vulnerabilities have been investigated globally, Southeast Asia (SEA) presents unique ecological, social, and political contexts that necessitate focused investigation. The objective of this scoping review is to identify, categorize, and map the key vulnerability factors that influence the risk of malaria infection among communities in malaria-endemic regions of Southeast Asia. Adopting a social determinants of health (SDH) framework, the review evaluates the influence of environmental, socioeconomic, and other variables on malaria vulnerability. Vulnerability is not merely a biological risk, but rather a consequence of systemic exclusion, weak health systems, environmental exposure, and limited access to services [21]. Synthesizing current evidence through this lens underscores the complex and intersecting drivers of risk, reinforcing the need for equitable, multisectoral, and context-specific strategies to support malaria elimination and health equity in the region.

## Methodology

### The review protocol

This scoping review was conducted following the PRISMA-ScR (Preferred Reporting Items for Systematic Reviews and Meta-Analyses Extension for Scoping Reviews) guidelines, which consist of 22 essential checklist items to ensure transparency and methodological rigour [22]. The review process was methodically integrated with key elements taken from PRISMA-ScR, including the study selection, data extraction, goals, eligibility criteria, search strategy, and synthesis of the results (see Additional file 1). A research question was developed to explore the study gaps on malaria in SEA region. The guiding question of this review is: what are the key vulnerability factors influencing the risk of malaria infection among communities in malaria-endemic regions of Southeast Asia? The population, exposure and outcome (PEO) framework of the current study were established as follows:

- P: Communities in SEA, particularly those in malaria-endemic regions, including individual, family, and communities level.
- E (Exposure): Risk factors and vulnerabilities, defined as increased exposure to malaria and limited capacity to avoid, prevent, or manage the disease. This includes socioeconomic, geographic, behavioural, and health system-related factors that influence both infection risk and access to malaria-related services.
- O (Outcome): Variations in malaria infection and transmission, including incidence, prevalence, and reported access to diagnosis, treatment, or prevention services.

### Data sources and search strategy

The literature search in this study was conducted from 20 October 2024 to 20 November 2024. The search strategy was conducted using three major English databases that include PubMed, Scopus, and Web of Science (WOS) to explore and identify potentially relevant studies that reported risk factors and vulnerabilities among SEA countries towards malaria, between January 2014 and October 2024. The search strategy combined keywords and Boolean operators to capture articles containing terms related to “malaria”, “vulnerability”, “risk factors”, and the names of Southeast Asian countries, which include: Brunei, Cambodia, East Timor (Timor-Leste), Indonesia, Laos, Malaysia, Myanmar, the Philippines, Singapore, Thailand, and Vietnam. Table 1 displays the particular search string created for this purpose.

**Table 1:** Search string for review

Database	Search String
Scopus	TITLE-ABS-KEY (("vulnerabilities" OR "vulnerability" OR "risk" OR "risk factors" OR "factors" OR "determinant" OR "determinants" OR "exposure" OR "exposures" OR "susceptibility" OR "susceptibilities" OR "susceptible") AND ("malaria" OR "plasmodium") AND ("individual" OR "family" OR "families" OR "community" OR "communities" OR "society" OR "societies" OR "neighborhood" OR "neighbourhood" OR "person" OR "people") AND ("southeast asia" OR "south east asia" OR "malaysia" OR "indonesia" OR "brunei" OR "singapore" OR "philippines" OR "thailand" OR "myanmar" OR "lao pdr" OR "laos" OR "vietnam" OR "cambodia" OR "timor leste"))
Web Of Science	ALL (("vulnerabilities" OR "vulnerability" OR "risk" OR "risk factors" OR "factors" OR "determinant" OR "determinants" OR "exposure" OR "exposures" OR "susceptibility" OR "susceptibilities" OR "susceptible") AND ("malaria" OR "plasmodium") AND ("individual" OR "family" OR "families" OR "community" OR "communities" OR "society" OR "societies" OR "neighborhood" OR "neighbourhood" OR "person" OR "people") AND ("southeast asia" OR "south east asia" OR "malaysia" OR "indonesia" OR "brunei" OR "singapore" OR "philippines" OR "thailand" OR "myanmar" OR "lao pdr" OR "laos" OR "vietnam" OR "cambodia" OR "timor leste"))
PubMed	("vulnerabilities"[All Fields] OR "vulnerability"[All Fields] OR "risk"[All Fields] OR "risk factors"[All Fields] OR "factors"[All Fields] OR "determinant"[All Fields] OR "determinants"[All Fields] OR "exposure"[All Fields] OR "exposures"[All Fields] OR "susceptibility"[All Fields] OR "susceptibilities"[All Fields] OR "susceptible"[All Fields]) AND ("malaria"[All Fields] OR "plasmodium"[All Fields]) AND ("individual"[All Fields] OR "family"[All Fields] OR "families"[All Fields] OR "community"[All Fields] OR "communities"[All Fields] OR "society"[All Fields] OR "societies"[All Fields] OR "neighborhood"[All Fields] OR "neighbourhood"[All Fields] OR "person"[All Fields] OR "people"[All Fields]) AND ("southeast asia"[All Fields] OR "south east asia"[All Fields] OR "malaysia"[All Fields] OR "indonesia"[All Fields] OR "brunei"[All Fields] OR "singapore"[All Fields] OR "philippines"[All Fields] OR "thailand"[All Fields] OR "myanmar"[All Fields] OR "lao pdr"[All Fields] OR "laos"[All Fields] OR "vietnam"[All Fields] OR "cambodia"[All Fields] OR "timor leste"[All Fields])

The searches were applied to titles and abstracts using controlled vocabulary (e.g., MeSH terms in PubMed) and keyword variations. Search results were imported into EndNote™ 20, a reference manager used to organize citations, automatically detect and remove duplicates, and streamline the screening process.

### Eligibility criteria

Included studies were original research published from 2014 to 2024, concentrating on vulnerabilities to malaria infection in SEA populations. Only studies focusing on SEA communities as the population and malaria as the primary outcome were included. Non-original works such as reviews, commentaries, books, reports, and conference proceedings were excluded from consideration. Research on additional vector-borne diseases, including dengue and filariasis, was also excluded. Articles published in languages other than English were considered only if full-text English translations were publicly available on the publisher’s website or accessible through institutional databases. This decade-long period guarantees the incorporation of current and pertinent evidence.

### Study selection

All titles and abstracts were screened independently by two reviewers using the agreed inclusion and exclusion criteria. Studies that reported malaria cases and related risk or vulnerability factors were selected for full-text review. Then, the full-text articles were checked by three reviewers. Each article was reviewed by at least two people, and the third reviewer helped confirm the decision or stepped in when the results were unclear. Disagreement regarding the fulfilment of study criteria or the interpretation of specific details were settled through discussion until consensus was achieved. Two reviewers independently charted data using a standardized data extraction form piloted on five studies to ensure consistency and clarity. The process was duplicated to reduce bias and improve reproducibility. The extracted data were organized into three sections: (1) general study details, (2) study design, population, and methodology, and (3) key findings and outcomes. Since the purpose of this review was to map the breadth and scope of the body of current literature rather than evaluate the risk of bias or the quality of the evidence, a formal critical appraisal of individual studies was not carried out in accordance with its aims.

## Results

**Fig. 1:**
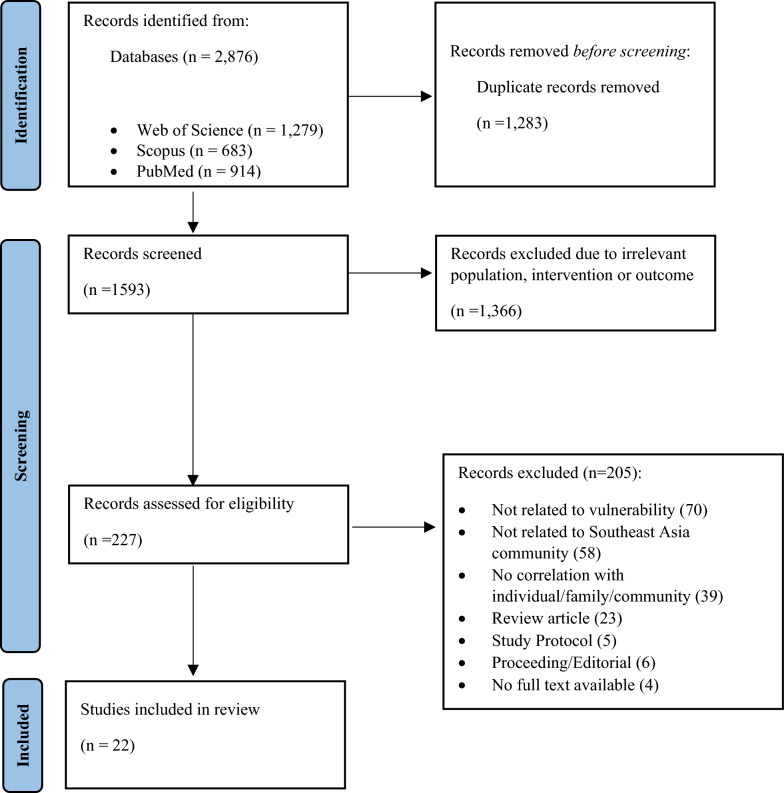
The PRISMA flow diagram

The literature search across three databases—Web of Science (1279), PubMed (914), and Scopus (683)—yielded 2876 articles (Fig. 1). After removing 1283 duplicates, 1593 articles remained for title and abstract screening. Of these, 1366 were excluded for not meeting inclusion criteria based on title, population, exposure, or outcomes. Full-text screening was conducted on 227 articles, with 22 studies published between 2015 and 2024 ultimately included in this review (Table 1). The selected studies covered several Southeast Asian countries, including Malaysia (*n* = 6), Indonesia (*n* = 6), Myanmar (*n* = 2), and one study each from the Philippines, Vietnam, and Cambodia. Additionally, five studies addressed malaria-related issues in cross-border regions. The research designs varied, comprising 12 cross-sectional studies, eight case–control studies, one cohort study, and one mixed-methods study. Most studies focused on rural and endemic communities, with two specifically investigating migrant populations. This geographical and methodological diversity provides a comprehensive view of malaria vulnerability in the region. Thematic synthesis was conducted using an inductive approach. Key findings were extracted, coded, and grouped based on conceptual similarities to identify overarching vulnerability domains. This process resulted five key domains: biological influences, demographic and socioeconomic parameters, built and lived environments, behaviour and practices, and access to healthcare services and information. These domains were shaped by iterative reading of the findings and discussions among reviewers to ensure conceptual coherence and thematic saturation. Notably, some studies reported unusually high odds ratios for certain risk factors which may reflect strong context-specific associations or methodological variations. Heterogeneity was evident across the included studies in terms of study populations, settings, definitions of exposures, and measured outcomes. This variation was taken into account during synthesis, and findings were reported narratively without meta-analysis, in accordance with scoping review methodology. Tables 2 and 3 summarize key characteristics and thematic findings from all included studies.

**Table 2:** The selected publication study location, design and population

No.	Author	Year	Country	Study design	Population
1	Hanandita and Tampubolon [23]	2016	Indonesia	Cross-sectional study with Bayesian hierarchical logistic regression	22,643 individuals from the National Basic Health Research 2007 from Papua or Irian Jaya, Indonesia
2	Gryseels et al. [24]	2015	Cambodia–Vietnam	Mixed method (Qual to Quan)	Male household heads and unmarried individuals Vietnam–Cambodia border
3	Cahyaningrum and Sulistyawati [25]	2018	Indonesia	Case–control study	Kaligesing Subdistrict, Purworejo District, Central Java Province, Indonesia, targeting adult residents. 96 individuals (48 cases and 48 controls)
4	Chen et al. [26]	2018	Myanmar–China	Cross-sectional study with stochastic simulation model (SSM)	Five villages along the Myanmar–China border in Yingjiang County, Yunnan Province, China
5	Matsumoto-Takahashi et al. [27]	2015	Philippines	Cross-sectional study	218 individuals from 20 rural villages in four endemic municipalities (Roxas, Puerto Princesa, Bataraza, and Brooke’s Point) of Palawan, Philippines
6	Munajat et al. [28]	2021	Malaysia	Cross-sectional study	536 people of indigenous Orang Asli community from five villages in Pos Kuala Betis, Gua Musang District, Kelantan, Malaysia
7	Ramdzan et al. [29]	2020	Malaysia	Cross-sectional study	Secondary data from 1,222 patients screened in 23 public health clinics in Sabah, Malaysia
8	Canavati et al. [30]	2019	Vietnam	Case–control study	Forest-goers in three communes of Dong Xuan district, Phu Yen Province, Vietnam. A total of 175 participants
9	Chin et al. [31]	2021	Malaysia	Retrospective, population-based case–control study	266 confirmed *Plasmodium knowlesi* (*P. knowlesi*) malaria cases and 532 matched controls, making a case-to-control ratio of 1:2. Native Sabahan
10	Hasyim et al. [32]	2019	Indonesia	Cross-sectional study	Data from the Basic Health Research (RISKESDAS) survey of 130,585 participants. Focused on five highly endemic provinces: Central Sulawesi, East Nusa Tenggara, Maluku, Papua, and West Papua
11	Soe et al. [33]	2017	Myanmar	Cross-sectional study	406 internal migrants working in gold mining, rubber, and oil palm plantations in three malaria-endemic townships of Myanmar: Shwegyin, Thanbyuzayat, and Kawthaung
12	Dunning et al. [34]	2022	Myanmar	Case–control study	119 malaria-positive cases and 1,744 control participants (malaria-negative) were recruited from 41 public health facilities in Ayeyarwady Region, Myanmar
13	Xu et al. [35]	2022	China-Myanmar	Case–control study	223 malaria cases (152 from China and 71 from Myanmar) and 446 control participants (304 from China and 142 from Myanmar) along China-Myanmar border population
14	Herdiana et al. [36]	2016	Indonesia	Cross-sectional study, utilizing both active and passive surveillance data	Population in Aceh Besar District, Indonesia, between 2014 and 2015 Passive surveillance: Malaria cases were detected from health facilities, where symptomatic patients were diagnosed through PCR-confirmed infection Active surveillance: 1495 community members were screened through reactive case detection (RACD),
15	Wang et al. [37]	2019	Thailand Myanmar	Cohort study	Kanchanaburi and Ratchaburi provinces, located along the Thailand–Myanmar border Involved 999 local individuals from these areas who were monitored for malaria risk factors
16	Rejeki et al. [38]	2021	Indonesia	Case–control study	138 malaria cases and 138 controls (non-malaria cases), with all malaria cases being clinically and laboratory-confirmed between January 1, 2015, and December 31, 2015 in Menoreh Hills, Java, Indonesia, specifically at the Primary Health Care in Menoreh
17	Grigg et al. [39]	2017	Malaysia	Case–control study	953 controls were selected, with 683 controls matched to *P. knowlesi* cases and 270 controls matched to non-*P. knowlesi* cases in Kudat and Kota Marudu, Sabah
18	Bryne et al. [40]	2023	Malaysia	Cross-sectional study	2015 population-based survey from northern Sabah, Malaysian Borneo
19	Doum et al. [41]	2023	Cambodia	Cross-sectional study	1301 participants of three high-risk populations which are forest rangers, forest goers and forest dwellers
20	Hasyim et al. [42]	2023	Indonesia	Case–control study	49 malaria cases and 49 malaria control cases from Primary Health Centres
21	Fornace et al. [43]	2019	Malaysia	Cross-sectional study	10,100 individuals sampled across 2849 households in 180 villages of northern Sabah
22	Fornace et al. [44]	2018	Malaysia–Philippines	Cross-sectional study	3003 individuals from four endemic communities in Malaysia (Limbuak, Pulau Banggi, and Matunggung, Kudat) and The Philippines (Bacungan)

**Table 3:** The selected publication findings summary

	Author	Significant findings*
1	Hanandita and Tampubolon [23]	Higher risk of malaria infection: • Elderly (55+ years) (OR 1.18, 95%CI 1.00–1.39) • Females had a slightly lower risk than males (4%) • Malaria risk was higher in lowland (OR: 2.99, 95%CI 1.84,4.59) compared to highland • High risk in a rural area (OR: 1.43, 95% CI 1.29–1.57) and in a densely forested district (OR: 1.08, 95% CI 1.00–1.17)
2	Gryseels et al. [24]	• Boys were more likely than girls to sleep unprotected (OR: 2.63, *p* = 0.05) • Higher proportion stayed overnight in the deep forest (OR: 6.61, *p* < 0.001) • Higher likelihood of using torn nets (OR: 3.63, *p* = 0.01)
3	Cahyaningrum and Sulistyawati [25]	Higher risk of malaria infection: • Not using bednets (aOR: 4.44, 95% CI 1.52–12.93) • Not closing doors and windows at night until morning (aOR: 6.46, 95% CI 2.30–18.12)
4	Chen et al. [26]	• Linear increase in malaria vulnerability with higher proportions of mobile populations (*χ*^2^ = 0.487, *p*: 0.485) • Living indoors offered just 5% additional protection compared to living outdoors
5	Matsumoto-Takahashi et al. [27]	• Patients living near a microscopist^#^ were more likely to receive appropriate treatment (aOR: 6.22, 95% CI 2.85–13.58) • Proximity to private pharmacies was associated with lower likelihood of appropriate treatment (aOR: 0.34, 95% CI 0.15–0.79) • Better knowledge of malaria symptoms was positively associated with participation in community awareness activities led by microscopists, increased self-awareness and timely care-seeking (Path Coefficient: 0.36 (*p* < 0.001) • Participants engaged in awareness activities had higher odds of appropriate treatment (aOR: 3.12, 95% CI 1.68–5.78)
6	Munajat et al. [28]	Higher risk of malaria infection: • Spending every night at home • Presence of monkey 500 m near house • Entry to nearby forest Knowledge discrepancy between age (19–40 vs. 6–18 years, OR: 3.5–4.0, *p* < 0.05)
7	Ramdzan et al. [29]	Risk: • Living in rural areas (aOR: 0.004; 95% CI 0.002–0.009) • Male gender (aOR: 0.023; 95% CI 0.012–0.047)
8	Canavati et al. [30]	Risk of malaria during after-dark activities: • Collecting water (aOR: 1.99; 95% CI 1.02–3.90) • Bathing in streams (aOR: 2.44; 95% CI 1.02–5.88) • Working after dark (aOR: 2.93; 95% CI 1.35–6.34)
9	Chin et al. [31]	Higher risk of malaria infection: • Men (aOR: 2.71, 95% CI 1.63–4.50) • Spent nights in the forest (aOR: 1.92, 95% CI 1.20–3.06) • Not using mosquito repellent (aOR: 2.49, 95% CI 1.36–4.56) • Prior malaria infection and the likelihood of contracting *P. knowlesi* (aOR: 49.34, 95% CI 39.09–78.32)
10	Hasyim et al. [32]	Higher risk of malaria infection: • Individuals aged 35–44 years (aOR: 1.58, 95% CI 1.39–1.80) • Employed individuals (aOR: 1.13, 95% CI 1.06–1.20) • Lack of knowledge about healthcare facilities (aOR: 4.18, 95% CI 1.52–11.45) • Not using preventive behaviour (aOR: 1.18, 95% CI 1.01–1.38) • Living in homes made of unimproved materials (aOR: 1.30, 95% CI 1.09–1.54) Protective factor: • Females were less likely to contract malaria compared to males (aOR: 0.91, 95% CI 0.87–0.96)
11	Soe et al. [33]	Higher risk of malaria infection: • Male migrants (OR: 1.84; 95% CI 1.22–2.77) • Went out at dawn (OR: 2.36; 95% CI 1.58–3.52) • Behavioral lapses, such as sleeping under torn bed nets or not consistently using bed net (OR: 2.00; 95% CI 1.21–3.30; OR: 2.02; 95% CI 1.15–3.52, respectively) • Alcohol consumption (OR = 2.71; 95% CI 1.73–4.26) • Did not attend malaria health talks (OR = 1.78; 95% CI 1.20–2.65)
12	Dunning et al. [34]	Higher risk of malaria infection was associated with: • Males (aOR: 1.8, 95% CI 1.2–2.9) • Worksites located in forests, particularly those involved in logging (aOR: 2.7, 95% CI 1.5–4.6), rubber plantations (aOR: 3.0, 95% CI 1.4–6.8) • Sleeping in the forest in the past month (aOR: 2.6, 95% CI 1.1–6.3) • Extended forest travel for durations of 3–14 days (aOR: 8.6, 95% CI: 3.5–21.4) or longer (aOR: 8.4, 95% CI 3.2–21.6)
13	Xu et al. [35]	Higher risk of malaria infection: • Overnight stays away from home (aOR: 13.37, 95% CI 6.32–28.28) • Staying overnight in rural lowland and foothill areas (aOR: 2.73, 95% CI 1.45–5.14) and staying overnight at altitudes < 500 m (aOR: 5.66, 95% CI 3.01–10.71) • Proximity to streamlets (≤ 100 m) (aOR: 9.98, 95% CI 4.96–20.09) • Lack of knowledge about malaria transmission (aOR: 2.17, 95% CI 1.42–3.32)
14	Herdiana et al. [36]	Higher risk of malaria infection: • Males (aOR: 12.5, 95% CI 3.0–52.1) • Adults aged 16–45 years (aOR = 14.0, 95% CI 2.2–89.6) • visited the forest in the previous month (aOR = 5.6, 95% CI 1.3–24.2) • People working near or in the forest, especially requiring overnight stays, [aOR = 7.9, 95% CI 1.6–39.7 (compared to those with workplaces not near or in the forest)] • Among those screened through RACD, febrile individuals were more likely to be malaria cases • Proximity to index case: cases were more likely to reside within 100 m of the index case
15	Wang et al. [37]	• Age was found to be a strong predictor, with adults being more likely to acquire P. vivax infections but less likely to experience clinical episodes (*p* < 0.05) • Seasonality (May to September) and previous clinical malaria history were important in predicting clinical episodes (*p* < 0.05) • Travel to Myanmar was identified as a key risk factor for acquiring malaria, particularly P. vivax (*p* < 0.05) • Indoor Residual Spraying (IRS) consistently appeared as a significant protective factor against malaria infections (*p* < 0.05)
16	Rejeki et al. [38]	Higher risk of malaria infection: • Houses located at an altitude greater than 500 m above sea level (OR: 3.62, 95% CI 1.61–8.16) • Houses made of bamboo/wood without insecticides (OR: 2.20, 95% CI 1.31–3.71) • Not spraying insecticides for past 1 year (OR: 2.08, 95% CI 1.09–3.95) • Houses located less than 100 m from mosquito breeding sites (OR: 1.93, 95% CI 1.03–3.64)
17	Grigg et al. [39]	Higher risk of malaria infection was associated with: • Age 15 years or older: aOR: 4.16, 95% CI 2.09–8.29, *p* < 0.0001 • Male gender: aOR: 4.20, 95% CI 2.54–6.97, *p* < 0.0001 • Plantation work: aOR: 3.50, 95% CI 1.34–9.15, *p*: 0.011 • Sleeping outside: aOR: 3.61, 95% CI 1.48–8.85, *p*: 0.0049 • Travel: aOR: 2.48, 95% CI 1.45–4.23, *p*: 0.0010 • Awareness of monkeys in the area (past 4 weeks): aOR: 3.35, 95% CI 1.91–5.88, *p* < 0.0001 • Having open eaves or gaps in walls: aOR: 2.18, 95% CI 1.33–3.59, *p*: 0.0021 • Farming occupation: aOR: 1.89, 95% CI 1.07–3.35, *p*: 0.028 • Clearing vegetation: aOR: 1.89, 95% CI 1.11–3.22, *p*: 0.020 • Long grass around the house: aOR: 2.08, 95% CI 1.25–3.46, *p*: 0.0048 4. Protective factors: • G6PD deficiency seemed to be protective against *P. knowlesi* infection: aOR: 0.20, 95% CI 0.04–0.96, *p*: 0.045 • Residual insecticide spraying of household walls: aOR: 0.52, 95% CI 0.31–0.87, *p*: 0.014 • Presence of young sparse forest: aOR: 0.35, 95% CI 0.20–0.63, *p* = 0.0040 • Rice paddy around the house: aOR: 0.16, 95% CI 0.03–0.78, *p*: 0.023
18	Bryne et al. [40]	Higher risk of malaria infection: • Higher travel times to hospitals and clinics (95% CI more than 1.0) • Various forest activities (95% CI more than 1.0) • Male gender (95% CI more than 1.0) • Falling within higher wealth quantiles was a protective factor for historic exposure to both *P. falciparum* and *P. vivax*, and the head of the household being educated to a secondary level or above was a protective factor for recent and historic exposure to *P. falciparum* (OR not explicitly provided)
19	Doum et al. [41]	• Female had less odds contracting malaria than males (aOR: 0.61, 95% CI 0.38–0.97, *p* < 0.05)
20	Hasyim et al. [42]	Risk: • Proximity to Mosquito Breeding Sites: Living within 100 m of mosquito breeding sites increased the risk of malaria (aOR: 3.88, 95% CI 1.67–8.97) Protective factors: • Mosquito Repellent: Usage reduced the risk of malaria by 71% (AOR: 0.29, 95% CI 0.11–0.64) • Wire Mesh on Ventilation: Installing wire mesh windows reduced the malaria risk by 76% (aOR: 0.24, 95% CI 0.10–0.57)
21	Fornace et al. [43]	Risk: • Age (per 10 years) (OR: 1.332, 95% CI 1.278–1.388) • Male sex (OR: 1.245, 95% CI 1.038–1.480) • Reported contact with macaques (OR: 1.419, 95 CI% 1.168–1.709) • Reported forest activities (OR: 1.871, 95% CI 1.447–2.368) • Irrigated farming fractal dimension (OR: 1.171,95% CI 1.065–1.282) • Proportion of pulpwood plantations (3000 m radius) (OR: 1.152, 95% CI 1.068–1.235) • Oil palm perimeter area ratio (3000 m radius) (OR: 1·101, 95% CI 1.006–1.198) Protective factors: • Insecticides usage (OR: 0.765, 95% CI 0.634–0.913) • House at ground level (OR: 0.760, 95% CI 0.632–0.906) • Elevation (per 1000 m) (OR: 0.481, 95% CI 0.290–0.738) • Intact forest perimeter–area ratio (5000-m radius) (OR: 0.857, 95%CI 0.752–0.961)
22	Fornace et al. [44]	Higher risk of malaria infection: • Age: 15–45 years (OR:2.05, 95%CI 1.30–3.22) 45–60 years (OR: 2.94, 95%CI 1.70–5.11) Over 60 years (OR: 2.46, 95%CI 1.32–4.58) • Farm or plantation work (OR:1.63, 95%CI 1.07–2.48) • Over 30% forest cover within 1km (OR: 2.40, 95%CI 1.29, 4.46) • Over 30% cleared/open area within 500m of house (OR: 2.14, 95%CI 1.35, 3.40)

^*^ All findings are significant to *p* < 0.05

^#^ Microscopists are trained community health workers (CHWs) specializing in malaria diagnosis and treatment in the Palawan province, Philippines. They play a critical role in providing accessible and effective healthcare for malaria, particularly in rural and resource-limited areas where formal health facilities are scarce

### Biological influences

A case–control study in Sabah, Malaysia, found that people with a history of malaria were 49 times more likely to get infected with *Plasmodium knowlesi (P. knowlesi)* compared to those without such a history (aOR: 49.34, 95% CI 39.09–78.32, *p* < 0.001) [31]. This supports similar findings from Thailand, where past malaria was a strong predictor of future episodes [37]. However, another study in the same area suggested that G6PD deficiency might protect against P. knowlesi infection (aOR: 0.2, 95% CI 0.04–0.96, *p* < 0.05) [39].

### Demographic and socioeconomic parameters

Eleven studies have examined the association between gender and vulnerability to malaria infection. Three studies reported that females had a lower risk of malaria [23, 32, 41], including findings by Hasyim et al., who demonstrated that females in five endemic provinces of Indonesia were significantly less likely to contract malaria compared to males (aOR: 0.91, 95% CI 0.87–0.96, *p* < 0.001) [32]. This trend was supported by similar findings from a study conducted in Cambodia [41]. Conversely, the majority of studies (*n* = 8) identified males as having a significantly higher risk of malaria, with odds ratios ranging from 0.02 to 12.5, underscoring gender as a key determinant of malaria susceptibility [29, 31, 33, 34, 36, 39, 40, 43].

Age was another factor highlighted in seven studies across Southeast Asia [23, 32, 36, 37, 39, 43, 44]. A cohort study by Wang et al. [37] found that adults were significantly more likely to contract *Plasmodium vivax* (*P. vivax*) (*p* < 0.05), a finding echoed by several other studies, which reported odds ratios for adult infection ranging from 1.58 to 14.0 [32, 36, 39]. Notably, one study from Indonesia indicated that individuals aged over 55 years were particularly vulnerable to malaria, further emphasizing age-related susceptibility [23].

Occupational exposure was identified in five studies as a significant risk factor [32, 34, 39, 43, 44]. Dunning et al. reported a strong association between malaria and work in logging and rubber plantations, with adjusted odds ratios ranging from 2.7 to 3.0 [34]. Similarly, a Malaysian case–control study found that individuals employed in plantations (aOR: 3.50, 95% CI 1.34–9.15, *p* = 0.011) and agriculture (aOR: 1.89, 95% CI 1.07–3.35, *p* = 0.028) were at elevated risk for P. knowlesi infection [39].

Higher wealth quantiles were protective factor for exposure to both *Plasmodium falciparum* (*P.* *falciparum*) and *P. vivax* in Bryne et al. study [40]. Additionally, having a household head with at least secondary education was associated with lower risk of both recent and past exposure to *P. falciparum.*

### Built and lived environment

Rural and forested areas have been consistently identified as high-risk zones for malaria transmission in nine studies [23, 24, 28, 29, 34, 36, 39, 43, 44]. In Malaysia, proximity to wildlife and forests was associated with increased risk, with factors such as frequent overnight stays at home, the presence of monkeys within 500 m, and forest entry contributing to vulnerability. Villagers who entered forests were 2.8 times more likely to encounter zoonotic malaria risks compared to those who stayed within the village (*p* < 0.01) [28]. In Myanmar, extended forest exposure for 3–14 days (aOR: 8.6, 95% CI 3.5–21.4) or longer (aOR: 8.4, 95% CI 3.2–21.6) significantly increased infection risk [34]. However, some environmental features, such as the presence of young sparse forests (aOR: 0.35, 95% CI 0.20–0.63, *p* = 0.004) and a high perimeter–area ratio of intact forest within a 5000-m radius, were associated with reduced malaria risk [44].

Geographical features, including landforms and elevation, were examined in four studies [23, 35, 38, 44]. Xu et al. [35] reported that staying overnight at elevations below 500 m (aOR: 5.66, 95% CI 3.01–10.71, *p* < 0.0001), as well as residing in lowland and foothill areas (aOR: 2.73, 95% CI 1.45–5.14, *p* = 0.0019), increased malaria risk. Proximity to streamlets—within 100 m—was also a significant risk factor (aOR: 9.98, 95% CI 4.96–20.09, *p* < 0.0001) [35]. A study in Indonesia found that houses located above 500 m elevation and near mosquito breeding sites also had elevated risk (OR: 3.62, 95% CI 1.61–8.16, *p* = 0.002) [38]. Conversely, higher residential elevation and houses built more than one metre above ground level were found to be protective against *P. knowlesi* exposure [44].

Seasonality also plays a role. One study reported that malaria incidence peaked between May and September, coinciding with the regional rainy season and optimal breeding conditions for mosquitoes [37].

Land use changes were implicated in two studies as contributing to increased malaria risk. Agricultural expansion—such as irrigated farming (OR: 1.171, 95% CI 1.065–1.282), pulpwood plantations (OR: 1.152, 95% CI 1.068–1.235), and oil palm cultivation (OR: 1.101, 95% CI 1.006–1.198)—was linked to higher *P. knowlesi* transmission, highlighting the influence of environmental modifications on vector-borne disease dynamics [43, 44].

Housing conditions were also identified as important determinants of malaria vulnerability in three studies [32, 38, 39]. In Indonesia, individuals living in homes constructed from unimproved materials had a significantly higher risk of infection (aOR: 1.30, 95% CI 1.09–1.54, *p* = 0.003) [32]. Rejeki et al. [38] found that bamboo or wooden houses lacking insecticide treatment were associated with increased malaria risk (OR: 2.20, 95% CI 1.31–3.71, *p* = 0.003). Protective factors included residual insecticide spraying on house walls (aOR: 0.52, 95% CI 0.31–0.87, *p* = 0.014) and the presence of rice paddies near the home (aOR: 0.16, 95% CI 0.03–0.78, *p* = 0.023) [39].

### Behaviour and practices

Protective behaviours against mosquito vectors were identified as important factors influencing malaria vulnerability in six studies across Southeast Asia. Common risk-enhancing behaviours included not using bed nets, sleeping outdoors or in forests, and poor use of repellents or insecticides [24, 25, 31, 32, 38, 39]. For example, in Indonesia, individuals who did not use bed nets had significantly higher odds of infection (aOR: 4.44, 95% CI 1.52–12.93, *p* = 0.006), and those who left doors and windows open overnight had a similarly increased risk (aOR: 6.46, 95% CI 2.30–18.12, *p* < 0.001) [25]. Forest sleeping and lack of mosquito repellent use were also associated with higher odds of P. knowlesi infection (aORs: 1.92 and 2.49, respectively) [31].

Two studies highlighted the protective role of insecticide use [37, 39]. Indoor residual spraying (IRS) was shown to significantly reduce malaria risk in cohort studies along the Thailand–Myanmar border and in Malaysia (aOR: 0.52, 95% CI 0.31–0.87, *p* = 0.014) [37, 39].

Seven additional studies examined behavioural factors, such as nighttime outdoor activities, mobility, and alcohol use [26, 30, 33–37]. Activities like fetching water or bathing after dark increased malaria risk (aORs: 1.99–2.93) [30], while forest sleeping (aOR: 2.6, 95% CI 1.1–6.3) [34] and high mobility were also linked to elevated vulnerability [26]. Alcohol consumption was associated with a higher malaria risk (OR: 2.71; 95% CI 1.73–4.26; *p* < 0.001), possibly due to reduced adherence to protective measures [33].

### Access to healthcare services and information

Limited access to malaria-related information and healthcare services contributes to increased vulnerability, as reported in six studies [27, 28, 32, 33, 35]. In Indonesia, poor knowledge of healthcare facilities was associated with a fourfold increase in malaria risk (aOR: 4.18, 95% CI 1.52–11.45, *p* = 0.005) [32]. Furthermore, participants engaged in awareness activities had higher odds of appropriate treatment [27]. Similarly, in Myanmar, individuals who did not attend malaria health talks had higher odds of infection (OR: 1.78, 95% CI 1.20–2.65, *p* = 0.004).

Four studies also highlighted healthcare access disparities [27, 33, 40]. In the Philippines, living near trained community microscopists significantly increased the likelihood of appropriate treatment (aOR: 6.22, 95% CI 2.85–13.58, *p* < 0.001), whereas proximity to private pharmacies was linked to reduced treatment quality (aOR: 0.34, 95% CI 0.15–0.79, *p* < 0.01) [27]. In Sabah, Malaysia, longer travel times to health facilities were associated with increased malaria risk [40].

## Discussion

### Comparing determinant of malaria infection across the world

Vulnerability to malaria in SEA is intricately shaped by a range of factors that fall under the umbrella of determinants of health. The biological, demographic, environmental, behavioural, and healthcare-related components interact in ways that influence the region’s malaria susceptibility and severity. By taking into account the individual, community, and larger social settings, these levels enable a comprehensive understanding of the variables that increase susceptibility to malaria. Comprehending these elements necessitates a thorough investigation of their respective roles in exacerbating and mitigating the risks of malaria transmission.

Understanding the significance of biological elements in malaria vulnerability is essential for comprehending regional differences in susceptibility, especially in SEA, where host genetic variation and Plasmodium species complexity have a major impact on malaria risk. The results of this review demonstrate the possibility of a dual effect of immune priming and activation of inflammatory mediators in people who have already had malaria, which could make them more vulnerable to zoonotic infections like *P. falciparum* as well as other types of malaria [45]. The correlation between previous exposure to malaria and subsequent infection is well-established worldwide, particularly with regard to *P. falciparum* [46]. Repeated exposure to *P. falciparum* usually results in some degree of immunity in the individual, which lessens the severity of subsequent infections. However, protection against other Plasmodium species, such as *P. vivax* and *P. knowlesi*, is not always provided by this acquired immunity [47]. It is worthy to note that the protective effect of glucose-6-phosphate dehydrogenase deficiency (G6PD) deficiency, which has been seen in SEA, is relevant worldwide. With a high prevalence in SEA, sub-Saharan Africa, and parts of the Mediterranean, this genetic characteristic is most common in areas where malaria is endemic. G6PD deficiency is seen in these regions as a form of balanced polymorphism, where the gene confers an advantage in areas where malaria is endemic by lowering the risk of developing severe malaria [48].

Males and age-related vulnerabilities have also been found to be at a higher risk of infection in most studies. The reason for this gender and age difference could be linked to behavioural and occupational factors. Outdoor jobs and activities like farming, logging, forest labour, and rubber tapping are more common among men, and they expose them to more malaria vectors, especially during the hours when mosquito bites are most common [49]. In forested and agricultural regions of Southeast Asia and sub-Saharan Africa, these exposure patterns notably increase malaria incidence in adult males [17]. Besides behavioural factors, biological and immunological differences may also contribute significantly. Research indicates that hormonal fluctuations, particularly testosterone levels, may influence immune responses to malaria, potentially increasing susceptibility in males [50]. However, in the majority of malaria-endemic areas, pregnant women and children—who are especially at risk because of their immature and weakened immune systems—face significant risks [49, 51, 52]. In a similar vein, wealth and occupation have a significant impact on malaria sensitivity and exposure. Rural, agricultural, and forestry workers in SEA are at high risk, mirroring trends in sub-Saharan Africa [53]. Socioeconomic disparities in malaria risk are a common thread globally, with wealthier populations typically experiencing lower exposure and better health outcomes due to better access to healthcare, mosquito control measures, and sanitation [17].

The built and lived environments have a significant impact on malaria transmission because they determine the existence of mosquito breeding grounds, human–vector interactions, and the dynamics of the disease as a whole. An individual's risk of acquiring malaria is greatly influenced by the environment, living conditions, and geography in SEA, where the disease is still endemic in many lowland, rural, and forested locations. Due to ideal breeding conditions in stagnant water, forests and rural areas frequently have high mosquito populations, which promotes cycles of zoonotic and human transmission [54]. In SEA, the existence of reservoirs like monkeys and other animals is comparable to the zoonotic threats in around the world, where diseases are becoming more well acknowledged as a factor in the spread of malaria in some areas [55]. The risk of malaria in the area is enhanced by geographic characteristics, including elevation, landforms, and proximity to water sources, when paired with environmental changes. [54, 55]. Similar trends have been noted worldwide, especially in highland areas of regions where malaria is endemic. For example, lowland and valley areas that offer ideal conditions for mosquito breeding increase the risk of malaria in parts of Ethiopia [56]. In Nepal, climate-driven changes have extended malaria transmission zones into formerly low-risk hilly and mountainous regions, like Morang and Dhangadi, suggesting increased susceptibility to environmental change [57]. On the other hand, because of their milder temperatures, which are less favourable to the mosquito life cycle, highland regions like Kenya often have lower rates of malaria transmission [58]. However, seasonal fluctuations in malaria transmission are widespread throughout the world, with periodic surges occurring in lowland areas, particularly during the rainy season when standing water offers mosquitoes perfect hatching grounds [59]. The quality of housing has a significant impact on the risk of acquiring malaria. The use of ITNs with IRS has been shown to be a very successful technique in lowering malaria cases in Africa, where indoor mosquito vectors are a primary mechanism of transmission, especially in areas with substandard housing conditions [60]. The efficiency of these treatments in enhancing housing conditions has been acknowledged in Asia, especially in remote and rural areas where access to healthcare and preventative measures may be restricted [61].

In SEA, behavioural variables play a major role in the transmission and spread of malaria, especially through individual and group behaviours that affect interactions between human vectors. Norms, belief, habits and custom affect the way of behaving in a population. The findings of this review demonstrate the proven value of personal protective equipment (PPE) in preventing malaria, including bed nets and protecting homes to limit indoor mosquito exposure. The extensive use of ITN has been a crucial intervention in lowering the incidence of malaria in sub-Saharan Africa [5]. But in SEA, a concerning trend shows that preventative measures such as using bed nets and repellent are either infrequent or not commonly used. Similar trends have been seen throughout the world, where collecting water and working in agriculture in the evenings are frequent activities that greatly increase the risk of contracting malaria, particularly in open and unprotected areas [62]. Additionally, migratory behaviour raises the risk of malaria transmission by introducing new vectors or facilitating the spread of infection across different regions. All these behaviours influence and interplay with socioeconomic and demographic factors, such as rural living, low income, and lack of education, which limit access to prevention tools and increase exposure to malaria vectors. Local health beliefs and customs should also be taken into account, as they have the potential to either exacerbate or mitigate the effects of the condition. None of the studies included touch on this aspect. Misconceptions regarding the causes and spread of malaria are common in several cultures, particularly in rural and isolated locations. These include misconceptions that malaria is spread by contaminated air or abnormalities in body humours, which make people shun contemporary preventative methods like indoor spraying or bed nets treated with insecticides [63, 64]. Malaria can be influenced by certain practices pertaining to home design, water supply handling, and hygiene [63, 64].

The vulnerability of SEA population to malaria is mostly caused by disparities in the accessibility and availability of healthcare and information services. In a region where malaria transmission is already high, these obstacles—which are frequently connected with demographic, socioeconomic, and environmental factors—make efforts to control the disease even more difficult. The results of this review demonstrate how the vulnerabilities towards malaria are increased by restricted access to healthcare practitioners and information. Particularly when it comes to informational gaps that cause treatment delays and the inability to take preventive action, marginalized people are extremely vulnerable. The problem of information availability has been acknowledged on a global scale as a critical factor in malaria control, particularly in environments with limited resources. In sub-Saharan Africa, where malaria remains endemic, community-based health education has been central to reducing malaria transmission. Community mobilization—whether through neighborhood health workers or community-led projects—significantly boosts the adoption of preventative measures like bed nets and encourages early diagnosis and treatment [65, 66]. However, migratory communities in Africa and other locations, like those in SEA, have limited access to healthcare services and health information, which frequently results in higher rates of morbidity and mortality [49]. For example, Timor-Leste experienced a resurgence of malaria infections in 2020 due to COVID-19-related disruptions in cross-border health services with eastern Indonesia. This setback demonstrates how external shocks, such as pandemics, can impede malaria control advancements, particularly in regions where health systems are vulnerable and reliant on regional collaboration [67]. Access to healthcare is also frequently hampered by poverty, isolated living circumstances, and inadequate infrastructure in regions of Africa, Asia, and South America where malaria is endemic [5]. High rates of malaria and increased mortality have resulted from rural communities in many African countries lacking access to health services. The World Health Organization has identified the provision of local health services and improving infrastructure as key strategies to tackle malaria, emphasizing the need for community health workers and mobile health units in remote areas [5, 65].

### Limitations of the included studies and the review process

The number of infections from community-based malaria clinics, regional public health offices, the national illness registration system, active and passive case detection, questionnaires, and screening were used in these reviewed studies to determine the incidence and malaria risk in humans. However, many of the included studies were constrained by incomplete or delayed national reporting systems, particularly in hard-to-reach or conflict-affected regions. These observations, as drawn from the studies reviewed, reflect broader systemic challenges such as underreporting, limited entomological monitoring, and fragmented cross-border surveillance, especially in areas with migratory or mobile populations [68–70] These challenges are consistent with known regional surveillance limitations and highlight the need for more integrated data systems that incorporate entomological and geographic risk mapping [71, 72]. This is because the malaria vector varies throughout the SEA region [72]. Defining the vulnerabilities based on vector density may shed some light on true malaria risk.

This review's methodology has several limitations that must be acknowledged. The review employed a rigorous and transparent search process across three major databases; however, the exclusion of grey literature and non-English articles lacking available translations may have resulted in the omission of pertinent findings. Secondly, the absence of critical appraisal of individual study quality aligns with scoping review methodology; however, this limitation hinders the assessment of the strength of evidence from each study. Third, while we utilized duplicate screening and data charting, the diversity of topics among the included studies may have resulted in heterogeneity that complicates direct comparisons of findings. Despite its limitation, this review offers a valuable synthesis of the multi-level factors affecting malaria vulnerability in Southeast Asia, structured within a determinants of health framework. This study maps existing evidence and identifies key research gaps, thereby advancing the discourse on targeted, equity-focused malaria interventions in the region.

## Conclusion

SEA vulnerability to malaria infection is multifaceted and firmly rooted in a network of intricately connected elements. Each of these elements—which include biological, demographic, socioeconomic, ecological, behavioural, and healthcare system-related factors—contributes in a different way to the disease's continued incidences in the area. The variation in malaria transmission dynamics across SEA countries further complicates efforts to combat the disease. The heterogeneity of malaria transmission dynamics across SEA underscores the need for tailored interventions rather than a one-size-fits-all approach. Therefore, it is crucial to incorporate ecological, socioeconomic, and healthcare system factors into malaria control strategies. Success requires a detailed strategy that targets the unique vulnerabilities in every region. In the end, addressing these vulnerabilities through all-encompassing, context-specific techniques that go beyond conventional tactics will be necessary in order to sustain the momentum of malaria elimination efforts in SEA. In order to effectively address these vulnerabilities, malaria control efforts need to take into account the wider determinants of health in addition to conventional interventions. While not the main focus of this review, our results highlight that addressing upstream risk factors like poor living environment, occupational exposure, and limited access to healthcare requires multi-sectoral collaboration, including engagement with communities, health sectors, and policymakers. In line with evidence from the included studies, strengthening local health systems, improving surveillance, and increasing access to preventive tools and accurate health information are crucial steps toward reducing malaria burden in these populations. By addressing the vulnerabilities associated with the socioeconomic, ecological, and health systems at the same time, SEA can keep reducing its incidence of malaria and eventually get closer to achieving malaria-free status.

## Supplementary Information

Supplementary Material 1.

## Data Availability

No datasets were generated or analysed during the current study.
